# Prevalence and Center Variability of Catheter-Based Hemodialysis in Vienna: Insights from the Vienna ACTS NOW Study

**DOI:** 10.3390/jcm13226733

**Published:** 2024-11-08

**Authors:** Markus Plimon, Maria-Elisabeth Leinweber, Amun G. Hofmann, Sara H. Ksiazek, Fadi Taher, Johannes Werzowa, Marcus Säemann, Afshin Assadian

**Affiliations:** 1 Department of Vascular Surgery, Clinic Ottakring, 1160 Vienna, Austria; 2 Department of Medicine VI, Clinic Ottakring, 1160 Vienna, Austria; 3 Department of Medicine I, Hanusch Clinic, 1140 Vienna, Austria

**Keywords:** vascular access, quality of care, resource management, hemodialysis, vascular surgery, end-stage renal disease

## Abstract

**Objectives**: The choice of vascular access continues to be a critical component in the management of hemodialysis patients. Despite the international consensus favoring arteriovenous (AV) fistulas, the use of central venous catheters (CVCs) remains prevalent, with substantial variations across countries and even among dialysis centers within the same region. This study examines the prevalence of CVC use among chronic hemodialysis (CHD) patients in Vienna, Austria, and explores inter-center differences. **Methods**: A cross-sectional analysis was conducted on patients receiving CVC-based CHD in Vienna as of March 2023. Patient demographics, comorbidities and their hemodialysis history were collected. Additionally, a subset of the population underwent vascular access (VA) mapping to assess eligibility for AV fistula (AVF) or AV graft (AVG) creation. **Results**: A total of 335 patients received CVC-based hemodialysis, equaling a CVC proportion of 42.5%. 191 (57.0%) patients on CVC-based CHD gave their consent to record their clinical data and vascular access history. Of the 191 included patients, 61 gave their consent to receive VA mapping. Of the 61 patients who received VA mapping, 60 (98.4%) were eligible for an upper extremity AVF or AVG. There was no significant difference regarding patient demographics, dialysis vintage, history of previous AVF or AVG or Charlson Comorbidity Index between the mapping and non-mapping group. The odds ratio of having a CVC in the absence of in-house vascular surgery was 3.41 (95% CI: 2.31–5.02, *p*-value < 0.001) compared to patients with in-house vascular surgery. **Conclusions**: The majority of patients that consented to ultrasound VA mapping fulfilled vascular requirements for AVF or AVG creation. Our study highlights the potential to decrease the prevalence of CVC-based CHD in Vienna that could translate to a reduction in CVC-associated complications.

## 1. Objectives

Chronic kidney disease (CKD) is a global public health issue of growing concern, responsible for 1.2–1.3 million deaths in 2017. The mortality rate from CKD increased by 41.5% between 1990 and 2017, underscoring the urgent need for effective treatment strategies, including kidney replacement therapies (KRTs) such as chronic hemodialysis (CHD) [1]. CHD is a life-sustaining therapy for patients with end-stage renal disease (ESRD), but the success of CHD depends heavily on the functionality of the vascular access (VA). The choice of VA plays a pivotal role in the treatment of affected patients, impacting both short-term outcomes as well as long-term patient quality of life and wellbeing. Several VA options exist, including central venous catheters (CVCs) and arteriovenous VAs that can be further differentiated into arteriovenous fistulas (AVFs) and arteriovenous grafts (AVGs). The choice of VA type is influenced by numerous factors, such as age, or comorbidities like diabetes mellitus and cardiovascular disease, all of which may negatively affect fistula maturation [2,3]. Rates of early failure within one month of VA creation are generally high for arteriovenous fistulas, even though reported numbers offer a wide range from 5 to 46% [4]. Other early/peri-operative complications include infection, especially in arteriovenous grafts, thrombosis, hemorrhages, seromas, and VA-induced ischemia [5,6].

Whenever feasible, autologous AVFs are generally preferred over AVGs or CVCs, as they may offer superior long-term patency and survival [7]. CVCs, however, feature distinct benefits and limitations. While they do not require a maturation phase and are therefore potentially advantageous in acute settings, they are associated with inferior long-term outcomes including a higher all-cause mortality. Central-line associated bloodstream infections (CLABSIs) are a significant contributor to increased morbidity and mortality rates. The continued use of CVCs is also associated with fibrin sheath formation and the thrombosis of central veins, possibly limiting future VA options. Consequently, the latest KDOQI guidelines recommend AV fistulas as the preferred vascular access, provided it aligns with the patient’s end-stage renal disease (ESRD) life plan and treatment goals [8]. Although there is still debate about the quantitative relationship between the type of vascular access and various clinical outcomes, the proportion of patients using catheter-based dialysis remains a key indicator of hemodialysis management quality within a healthcare system [9]. Despite this international consensus favoring AV fistulas, the prevalence of catheter use not only differs widely across countries but also among different dialysis facilities within the same country [10,11,12,13].

To quantify the prevalence of CVC-based dialysis among chronic hemodialysis patients in Vienna and to identify potential between-center variations, the prospective multicenter study “Vienna ACTS NOW—Vienna Vascular Access Studies on Exchange of Dialysis Catheters for Fistulas” was initiated in 2023. Vienna ACTS NOW aims to identify, and address disparities in VA practices among dialysis centers in Vienna, Austria, focusing on understanding the prevalence of, as well as the reason for, catheter-based HD. The study aims to investigate a potential inter-center variability and explores factors influencing CVC use, including the availability of vascular surgery expertise at the respective centers, patient demographics, and the extent of pre-emptive VA mapping.

There is a paucity of data on how access to VA expertise, and mapping opportunities and mapping protocols within dialysis centers might further influence or mitigate CVC prevalence. By identifying patients eligible for AVF creation who are currently dependent on CVCs, this study aims to highlight opportunities for reducing catheter use in Vienna with an aim to thereby lower CVC-associated morbidity. Vienna ACTS NOW not only seeks to document CVC prevalence but also to analyze underlying causes or reasons by assessing the process of treatment plan formulation for ESRD patients. The study further aims to inform strategies for uniform and potentially optimized VA practice across Vienna. Insights from this study are intended to address CVC-related questions that remain, in some cases, unanswered. It aims to contribute to a better understanding of CVC utilization for CHD in Austria in order to help improve the quality of care for ESRD patients.

## 2. Methods

Vienna ACTS NOW is a multi-center cross-section analysis investigating patients with catheter-based vascular access (VA) at 7 institutions providing CHD in Vienna. The Vienna ACTS NOW collaborators and participating centers are listed in the Acknowledgements section.

To estimate the number of potential participants in this study we accessed the data on VA modalities of the Austrian Dialysis and Transplant Registry for Vienna from 2013 to 2023.

Participating centers reviewed the inclusion and exclusion criteria for all patients undergoing dialysis at their center. All patients over 18 years of age who were on catheter-based CHD on 1 March 2023 were eligible for inclusion. Patients were provided information about the investigation by their treating nephrologists. Informed consent was obtained from all subjects involved in the study. The data sets were recorded using ePRO software provided by Castor (Amsterdam, The Netherlands). Collected data included basic demographic and clinical information such as age, sex, co-morbidities and dialysis history. Dialysis vintage was defined as the period between the initiation of dialysis and inclusion in the study. Participating patients were interviewed by their treating physicians to identify the reason for their VC-based hemodialysis. The treating physicians were tasked with documenting one main cause for catheter-based CHD for each participant. We calculated the Charlson Comorbidity Index as modified by Quan et al. [14].

Furthermore, patients were invited to participate in an ultrasound-based VA mapping. The VA mappings were mainly carried out by two highly experienced ultrasound examiners based in the same dedicated vascular access unit. One participating center opted to perform the VA mappings in-house by examiners with appropriate expertise. In all cases, the mappings were carried out according to a standardized VA mapping form provided by the study team. The threshold values for AVF or AVG creation were based on the 2018 European Society for Vascular Surgery VA guidelines [4].

To ensure data reliability, the study team routinely performed plausibility checks of all data sets. If the data were implausible or incomplete, queries were sent to the respective center for clarification.

### 2.1. Statistical Analysis

We conducted a descriptive statistical analysis and compared the means between the mapping and non-mapping groups using *t*-tests for continuous variables. Categorical variables are shown as frequencies and proportions. Testing for significant differences in proportion was carried out using the chi-squared test (two-tailed). Analyses were performed using SPSS Version 29.0 (IBM, New York, NY, USA).

### 2.2. IRB Approval

This study was authorized by the Ethics Committee of the City of Vienna, Austria (approval no. EK-22-104-VK, 28 November 2022), and adhered to the principles laid out by the Declaration of Helsinki. All participants were provided with detailed study information, and written informed consent was obtained prior to enrollment.

## 3. Results

**Figure 1:**
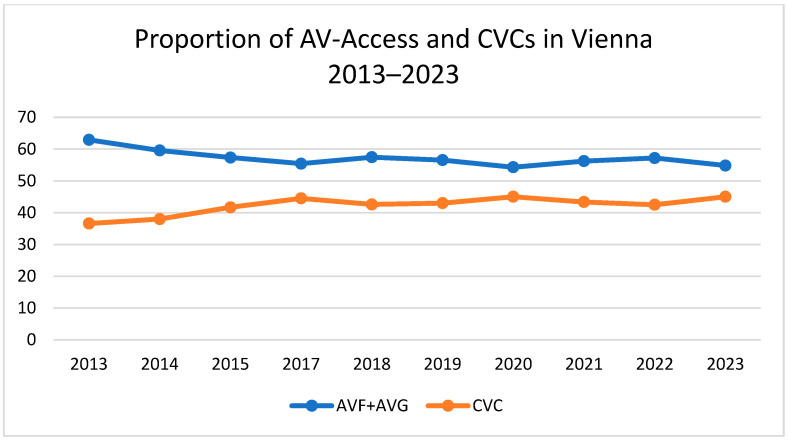
Proportion of arteriovenous access and central venous catheters in Vienna (years 2013–2023; data from 2016 unavailable).

The Austrian Dialysis and Transplant Registry (ÖDTR) recorded 1051 patients receiving CHD in Vienna during 2023 compared to 935 in the year 2013. Over the decade, the proportion of patients with arteriovenous fistulas (AVFs) or arteriovenous grafts (AVGs) declined (a combined total of 62.9% in 2013 vs. 54.8% in 2023), while the use of central venous catheters (CVCs) increased (36.6% in 2013 vs. 45.0% in 2023). The proportion of AVGs was stable over time (3.6% in 2013 vs. 4.4% in 2023). This shift in VA type prevalence is depicted in Figure 1. However, the data for 2016 were unavailable.

Seven of nine institutions providing CHD in Vienna, Austria, participated in this study, comprising a total of 789 patients on 1 March 2023. Six centers were hospital-based (hospital departments) with an associated out-patient service treating a total of 482 (61.1%) patients, while one center provided solely out-patient treatments for 307 (38.9%) patients. Of the total patient population, 335 patients (42.5% of all patients at the cut-off date for inclusion) received catheter-based CHD and were invited to participate in the study. Marked variability was observed across all participating centers, with CVC proportion per center ranging from 27.6% to 94.4% (see Table 1).

Of the 335 patients receiving CVC-based CHD, 191 (57.0%) consented to have their clinical data and vascular access history recorded, and 61 of these patients also consented to undergo vascular access (VA) mapping to allow an exploration of potential AVF or AVG eligibility.

The mean age of the included patients was 67.9 years (sd ± 15.1), and the median total duration of CVC-based CHD was 3.0 years (IQR ± 4.0). Of the 191 patients receiving catheter-based CHD participating in the study, 108 (56.5%) were male and 83 (43.5%) were female. Moreover, 73 (38.2%) patients had a history of either a previous AVF or AVG. In total, 15 (12.6%) patients had a history of kidney transplantation and 42 (22.0%) had a history of bacteremia.

Furthermore, 180 (94.2%) CVCs were placed in the internal jugular vein, 131 (72.8%) on the right and 49 (27.2%) on the left side. One CVC was located in the right and four in the left subclavian vein. Six patients had a femoral CVC. All CVCs in this study were tunneled catheters.

Of the six hospital departments, one center had access to an in-house vascular surgery department with a dedicated VA team and two centers had access to an in-house VA clinic occupied by individual vascular surgeons.

In the present patient cohort, the proportion of CVCs in centers with in-house access to vascular surgeons was lower than in those without in-house access to a vascular surgeon: CVC prevalence of 34.1% in centers with in-house vascular surgeons versus 63.8% in those with no in-house access to vascular surgeons (Table 2).

The odds ratio of having a CVC in the absence of an in-house vascular surgery department or service was 3.41 (95% CI: 2.31–5.02, *p*-value < 0.001) compared to patients cared for at a center with in-house vascular surgery.

Of the 61 patients who participated in VA mapping, 56 (91.8%) met the requirements for an upper extremity AVF. Four (6.6%) patients, who were not eligible for an AVF, met the requirements for an AVG. Only one patient (1.6%) did not meet the requirements for either AVF or AVG creation at an upper extremity according to ESVS guideline recommendations.

Various reasons were reported and recorded for continued CVC-based CHD. They are summarized by Table 3. “Bridge to destination” as a term was used to describe patients awaiting a kidney transplant or those with a maturing AVF (regarded as “time-limited” scenarios with short estimated remaining time on CVC-based CHD within the current analysis; 11.5% of the included patients). Among several other reasons, patients reportedly continued with CVC-based CHD due to factors such as fear of pain (11.0%), esthetic concerns (3.7%), prior AVF/AVG failures (16.2%), and organizational delays (3.7%). A large proportion of patients were reported to remain on CVC-based CHD due to their overall health status (17.3%). Notably, in 24.6% of patients, no clear reason could be determined for their continued CVC use.

**Figure 2:**
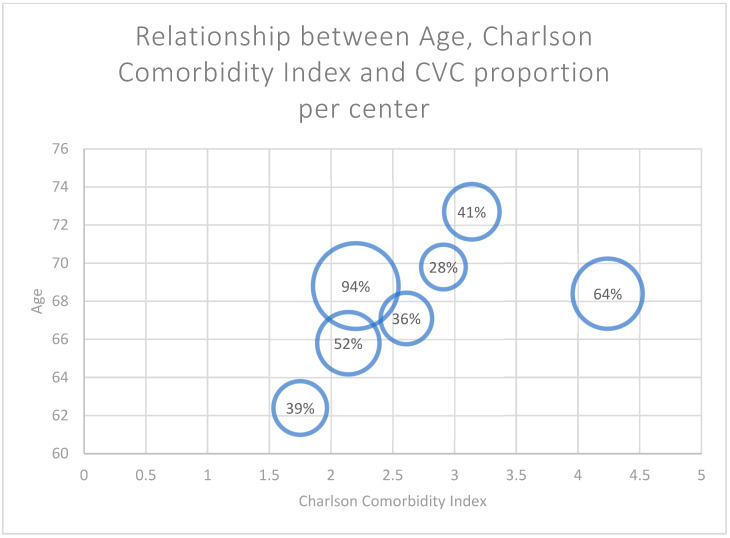
Relationship between age, Charlson Comorbidity Index and central venous catheter (CVC) proportion per center.

The Charlson Comorbidity Index as modified by Quan et al. was recorded for all 191 patients [14]. The relationship between the mean age (*y*-axis), the Charlson Comorbidity Index per center (*x*-axis), and the center’s catheter proportion (diameter of the blue sphere) is shown in Figure 2.

There was no significant difference between the mean age, dialysis vintage or Charlson Comorbidity Index between the mapping and non-mapping group. The percentage of previous AVF or AVG is described in the table below (Table 4).

## 4. Discussion

The 10-year ÖDTR data from 2013 to 2023 show an increase in CVC-based hemodialysis and a decrease in combined AVF and AVG-based hemodialysis in Vienna from 2013 to 2023. The most significant change happened between 2013 and 2017. CVC levels have since plateaued.

The creation and maintenance of AVFs and AVGs is time- and resource-intensive. Our study reveals a notable disparity in CVC usage across dialysis centers in Vienna, with factors such as in-house access to vascular surgery expertise correlating with significantly lower CVC prevalence. This finding highlights the importance of an integrated, multidisciplinary approach to HD patient management as centers with dedicated VA expertise included in our study seemed better positioned to transition patients from temporary CVCs to AVFs or AVGs. This observation and hypothesis align with the current KDOQI guidelines, which call for an individualized end-stage renal life plan for every patient formulated and maintained by a multidisciplinary team [8]. Thus, our findings underscore the need for collaboration among nephrologists, vascular surgeons, and dialysis nurses to enhance patient outcomes.

Our cross-sectional analysis of catheter-based CHD in Vienna highlights a significant variance in catheter proportions between the individual hemodialysis units, which is consistent with previous observations from other countries [12,15,16]. Most patients on CV-based CHD (61.8%) did not have a prior history of AVFs or AVGs. Additionally, 91.8% of patients participating in the VA mapping subgroup analysis met the criteria for AVF creation with an additional 6.6% eligible for AVG creation. Only one patient that participated in the VA mapping did not meet the mapping requirements for either an AVF or AVG as defined by the 2018 ESVS Vascular Access Guidelines [4]. This represents a significant number of patients who could be eligible for an AVF or AVG creation but were started on CV-based CHD for various reasons.

As depicted in Figure 2, the variance in CVC proportions per center was not dependent on reported mean age or Charlson Comorbidity Index in the current series. This finding may suggest that the difference in age and overall health status of the patient populations in the individual centers may not be the decisive factor that leads to the differences in CVC proportions. We hypothesize that factors beyond clinical eligibility—such as patient perceptions or resource availability—may contribute substantially to VA choices.

The reasons given for the continued use of CVCs in certain patients include fear of pain, esthetic concerns, previous VA failures, and organizational delays. This emphasizes a need for enhanced patient education and counseling to address patient preferences, fear, and prior experiences regarding AVF or AVG creation. Such improved counseling may alleviate concerns about pain and esthetics and could help inform patients about the risks and benefits of different VA types. Addressing patient concerns and improving patient education could facilitate more informed VA decisions and potentially reduce CVC reliance by helping to better align patient preferences with clinical recommendations.

Additionally, a substantial number of patients had no clear reason cited for their continued CVC use, suggesting that further investigation is needed to better understand and address potential barriers.

The high eligibility rate for AVF or AVG creation among mapped patients suggests that the routine use of VA mapping could help identify more patients who are suitable for permanent VA options.

The optimal timing for VA mapping remains difficult to determine. In order to balance the risk of the unnecessary placement of fistulae and the overuse of CVCs, the recent KDIGO guidelines call for VA planning if the risk of renal failure exceeds 40% in two years [17]. Implementing standardized mapping protocols could potentially lower CVC prevalence by promoting an earlier and more accurate identification of candidates for AVF or AVG creation. Even though fistula first is not necessarily a dogma to be enforced in every clinical scenario, the identification of patients in whom an alternative to CVC-based CHD can be offered comes with a prospect and potential benefit of lowering catheter proportions and associated complications.

This study has several limitations, the most important being selection bias. Patients with a long and possibly frustrating history of AV creation, revision and failure may be more likely to refuse study participation or ultrasound mapping. Additionally, physicians participating in the study may refrain from referring patients to VA mapping if they feel that their prior VA history makes the possibility for VA creation unlikely. Variability in CVC use between centers may have been influenced by factors that were not fully accounted for in the study, such as local policies, resource availability, or physician preferences—the potential influence of such center-specific practices cannot sufficiently be captured by the performed analysis. These center-specific factors could have contributed to the differences observed, and future research could aim to isolate these influences more effectively. The number of patients who participated in the study and the subsequent VA mapping remains rather low and affects performed subgroup analyses and their interpretation. Additional investigations into patient education practices on VA options might help mitigate personal and psychological barriers to AVF acceptance.

A high proportion of patients eligible for AVF or AFG creation in the mapping group and the lack of significant differences between the mapping and non-mapping groups in our recorded data may indirectly suggest an additional number of patients that have the technical prerequisites for AVF or AVG in the non-mapping group. At the very least, the high rate of technically feasible AVF or AVG creation options in the VA mapping group creates a realistic opportunity to lower catheter use in these patients. The findings from this study may indicate a recruitment problem, perhaps an opportunity for improved patient education, potentially a lack of access to resources for AV creation and the need for a more effective coordination of the existing resources.

## 5. Conclusions

This study underscores the considerable potential for reducing central venous catheter (CVC) use in chronic hemodialysis (CHD) patients across Vienna by exploring possibilities for early arteriovenous fistula (AVF) or graft (AVG) creation. While many patients on CVC-based hemodialysis included in the study fulfill the technical requirements for AVF or AVG creation, several factors—ranging from organizational barriers to patient concerns over pain and esthetics—contribute to the continued reliance on CVCs. Notably, institutions with access to in-house vascular surgery expertise demonstrated lower CVC prevalence in our study, emphasizing the importance of multidisciplinary collaboration in improving vascular access outcomes. The present study explores both clinical and psychosocial considerations of AVF and AVG eligibility. Our results potentially support routine vascular access (VA) mapping, enhanced patient education, and professional care from a multidisciplinary team of healthcare professionals as measures to better align patient preferences with current clinical recommendations.

CVCs, while certainly valuable and useful in acute or temporary contexts, also carry increased risks of infection, thrombosis, and long-term morbidity. While the authors do not advocate fistula first as a dogma to be enforced in every clinical scenario, reducing CVC prevalence in many ways aligns with broader goals to improve end-stage renal disease (ESRD) patient outcomes. Shifting to AVFs or AVGs where feasible could potentially lead to better outcomes for patients, but the effectiveness of such a shift, as our study demonstrates, likely depends on multiple factors.

Future studies may delve further into patient-specific and center-specific factors influencing VA decisions, potentially guiding tailored interventions that address the unique needs of patients and the resources available at different institutions. In particular, exploring patient education strategies could help reduce fears and misconceptions about AVFs, while ensuring patients are fully informed of the benefits and risks of the various available VA options. In combinations with enhanced access to VA expertise, such measures could support sustainable, high-quality dialysis care for patients with ESRD.

## Figures and Tables

**Table 1:** Center-specific benchmarks.

Center Code	Proportion of CVC	Recruitment	Proportion of Mapped Patients
1 *	27.6%	86.5%	62.5%
2	64.4%	72.4%	38.1%
3 *	36.0%	85.2%	0.0%
4	51.6%	45.8%	27.3%
5	94.4%	58.8%	30.0%
6	38.8%	30.3%	30.6%
7 *	41.4%	90.2%	27.0%

* Centers with in-house access to vascular surgeons.

**Table 2:** Proportion of CVCs in centers with and without access to vascular surgeons.

Vascular Surgeons	Number of Patients	Total Number of Catheters	Catheter Proportion
Yes	308	105	34.1
No	174	111	63.8

**Table 3:** Reasons given for CVC use.

	No Mapping	Mapping	Total
Fear of pain	14	7	21
Esthetic reasons	6	1	7
Prior AVF/AVG history	18	13	31
Out of vessels	7	0	7
Bridge to destination	17	5	22
Organizational delays	1	6	7
Overall health status	27	6	33
Patient preference	7	3	10
Cannulation problems	3	1	4
Other	1	1	2
None given	29	18	47
Total	130	61	191

**Table 4:** Comparison of mapping vs. non-mapping groups.

		n	Mean	SD	*p*
Age (in years)	Mapping	61	66.6	14.4	
	No Mapping	130	68.6	15.5	0.605
Dialysis vintage (in years)	Mapping	62	3.2	3.2	
	No Mapping	130	3.2	3.1	0.613
History of AVF or AVG	Mapping	61	38%		
	No Mapping	130	38%		
Charlson Comorbidity Index	Mapping	61	2.6	1.7	
	No Mapping	130	2.6	1.8	0.603

## Data Availability

The original contributions presented in the study are included in the article, further inquiries can be directed to the corresponding authors.
